# *Tsukamurella tyrosinosolvens *- An unusual case report of bacteremic pneumonia after lung transplantation

**DOI:** 10.1186/1476-0711-8-30

**Published:** 2009-11-12

**Authors:** Armelle Ménard, Sébastien Degrange, Olivia Peuchant, Thi Diem Tien Nguyen, Claire Dromer, Jeanne Maugein

**Affiliations:** 1 Laboratoire de Bactériologie - C.H.U. de Bordeaux, Hôpital Pellegrin et Hôpital du Haut-Lévêque - Place Amélie Raba-Léon - 33076 Bordeaux cedex, France; 2 Laboratoire de Bactériologie Université Victor Segalen Bordeaux 2, EA 3667, F33076 Bordeaux, France; 3 INSERM U853, F33076 Bordeaux, France; 4 Service de chirurgie thoracique, Hôpital du Haut-Lévêque, F33604 Pessac, France

## Abstract

**Background:**

Lung transplant recipients have an increased risk for actinomycetales infection secondary to immunosuppressive regimen.

**Case presentation:**

A case of pulmonary infection with bacteremia due to *Tsukamurella tyrosinosolvens *in a 54-year old man who underwent a double lung transplantation four years previously is presented.

**Conclusion:**

The identification by conventional biochemical assays was unsuccessful and *hsp *gene sequencing was used to identify *Tsukamurella tyrosinosolvens*.

## Background

Bacteria of the genus *Tsukamurella *are included in the order *Actinomycetales*, characterized by mycolic acids. Phylogenetic analyses have demonstrated that the genus *Tsukamurella *is related to the genera *Nocardia*, *Rhodococcus*, *Dietzia*, *Gordonia, Streptomyces, Corynebacterium *and *Mycobacterium *[1,2]. The name of *Tsukamurella *comes from Tsukamura, a microbiologist who in 1971 described the first strain of *Gordona aurantiaca*, isolated from sputum of a patient who was affected by a chronic lung pathology [3]. Some *Tsukamurella *species are commensal bacteria found in the environment, both at ground level and in water, and are opportunistic bacteria in patients with chronic pulmonary infection or in immunocompromized patients [4,5]. To date, *T. tyrosinosolvens *has been isolated only from human specimens and has always been associated with clinical disease [6].

In this report, we describe the first isolation of *T. tyrosinosolvens *from sputum and blood samples of a lung transplant patient.

## Case presentation

A 54-year old man was hospitalized in July 2008 for a pulmonary infection. His medical history indicated 1) a lung transplant in November 2004 for emphysema linked to an alpha-1-antitrypsin deficiency, 2) chronic respiratory insufficiency, and 3) hypertension. In addition, several infectious episodes were noted in 2005 and 2006 following a fracture of the left femoral neck in May 2005 with severe osteoporosis and a fracture of the right foot in June 2006.

Upon admission, the patient described a chronic cough with abundant expectorations during the previous ten days. No fever was noted. The bronchial endoscopy showed of the presence of abundant secretions in the right bronchial tree. The mucous was notably inflammatory. The bronchial biopsies revealed acute bronchitis signs. A broncho-alveolar fluid sample was sent to the bacteriology laboratory and the analysis showed 4 × 10^7 ^cells/mL with 93% polymorphs. The presence of Gram-positive bacilli was noted, but Ziehl-Neelsen staining was negative.

A few hours post- endoscopy, a fever appeared (temperature up to 40°C) and three series of aerobic and anaerobic blood cultures were done. Blood culture showed only Gram-positive bacilli from the three aerobic bottles after 5 days which were identical to those identified in the broncho-alveolar fluid grown at the same time.

A therapy associating imipenem and tobramycin led to a significant clinical improvement, with a reduction in sputum volume, cough and apyrexa within 3 days. A cardiac transesophagal echography was performed to rule out endocarditis. The evolution was favorable with an important decrease in the inflammatory syndrome, the C-reactive proteins decreased from 103 to 9.3 mg/L.

## Methods

With regard to the phenotypic identification, the isolate grew aerobically on blood agar at 37°C forming colonies with an unusual morphology. The colonies were rough and yellowish, similar to fungal growth. Phenotypic identification of Gram-positive organisms was performed employing the API Corynebacterium System (bioMérieux, Marcy l'Eoile, France) and other biochemical tests, including detection of arylsulfatase, lysozyme growth, and hydrolysis of casein, xanthine and tyrosine. They were all interpreted using conventional methods. The isolate was further examined using the GenoType Mycobacteria Molecular Genetic Assay (Hain Lifescience, Nehren, Germany); although both conjugate and universal controls were positive, the isolate failed to hybridize with the 13 bacterial species targetted by the kit. Antimicrobial susceptibility testing was carried out using a diffusion method. The strain was resistant to penicillin G, ampicillin, oxacillin, cefalotin, ceftriaxone, piperacillin/tazobactam, erythromycin, spiramycin and pristinamycin but was sensitive to imipenem, ertapenem, doxycycline, tigecycline, cotrimoxazol, rifampicin, vancomycin and linezolid. As identification based on this technology and the biochemical profile was unsuccessful, we turned to genotyping.

Concerning the genotypic identification, a 16S rRNA gene sequencing was first performed with the universal forward 27F (5'-AGAGTTTGATCMTGGCTCAG-3') and reverse 1492R (5'-TACGGYTACCTTGTTACGACTT-3') primers [7,8] generating amplicons of 1485 bp. PCR was performed with the Taq DNA polymerase (Promega, Charbonnières-les-Bains, France). Amplification parameters consisted of one cycle at 95°C for 5 min, followed by 35 cycles at 95°C for 30 s, 58°C for 30 s, and 72°C for 2 min, and finally one cycle at 72°C for 5 min. The products of PCR amplification were purified using MicroSpin S-400 HR columns (Amersham Pharmacia Biotech Inc., Uppsala, Sweden) and sequencing of these products was achieved on both strands using the initial set of PCR primers, and internal primers F1-16S-Seq-Univ (5'-GCTAACTCCGTGCCAGCAG-3') and R1-16S-Seq-Univ (5'-TTGCGGGACTTAACCCAAC-3'), using an Applied Biosystems 3130 *xl *Genetic Analyzer (Applied Biosystems, Foster City, CA, USA) with the fluorescent Big Dye terminator V1.1 Cycle Sequencing Kit (Applied Biosystems), according to the manufacturer's instructions. Amplified primer-less sequences were compared to the GenBank database with the nucleotide Blast program at the National Center for Biotechnology Information Computer server [9]http://blast.ncbi.nlm.nih.gov/Blast.cgi. The sequence showed 99% identity in a 1332 nucleotide overlap with *T. tyrosinosolvens *and the species identification was confirmed by using the bioinformatics bacterial identification tool BIBI [10] (strain Bx-62986 closely related to type strain *T. tyrosinosolvens* DSM 44234). Second, this species identification based on the 16S rRNA gene was confirmed with another identification based on heat shock protein coding gene sequencing. The available *hsp* sequences of *T. tyrosinosolvens *strain IMMIB D-1411 and *Tsukamurella paurometabola *strain 2061 (GenBank accession numbers: TTU90204 and AF352578), respectively were aligned using multiple sequence alignment with hierarchical clustering [11]http://bioinfo.genotoul.fr/multalin/multalin.html and analyzed to identify conserved regions for the primer design. The resulting primer set F2-HSP-Tsu (5'-AATTGCGTTCGACRAARAGG-3') and R2-HSP-Tsu (5'-AATCCATGCCACCCATGC-3') were designed using the web Primer3 software [12]http://frodo.wi.mit.edu/. Amplification parameters consisted of 95°C for 5 min, followed by 50 cycles of 95°C for 30 s and 60°C for 30 s, and 72°C for 90 s, and finally one cycle at 72°C for 5 min. Amplified DNA fragments were purified and direct sequencing was carried out on both strands (see above) with the PCR primers and the newly designed internal primers F3-HSP-Tsu (5'-GGACATGGCGATCCTCAC-3') and R3-HSP-Tsu (5'-ACGATGGTGGTCTCGTCCT-3'). When compared with the NCBI Blast program, the 1576 nucleotide sequence had 96% identity in a 1579 nucleotide overlap while the deduced protein sequence had a 97% identity in a 524 residue overlap, thus confirming the 16S rRNA gene identification.

The 16S rDNA and *hsp*65 sequences from the *T. tyrosinosolvens *Bx-62986 strain were submitted to GenBank and assigned accession numbers FJ643548 and FJ643549, respectively.

**Figure 1:**
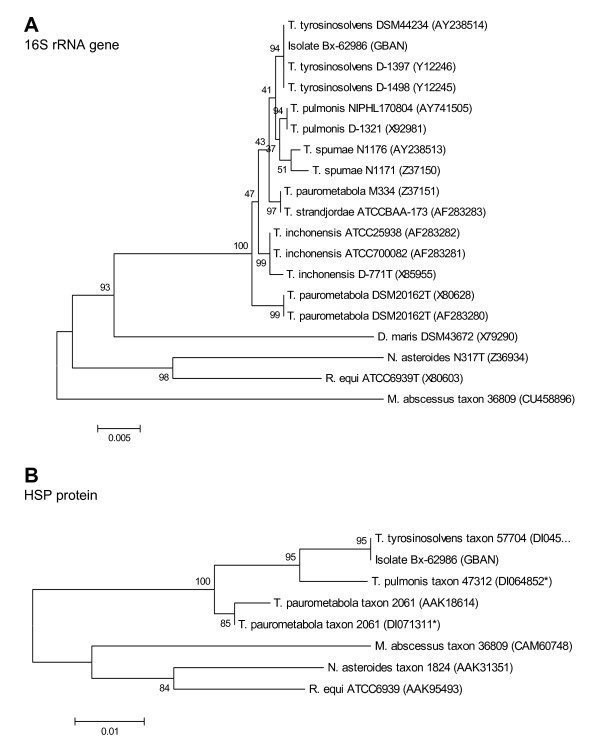
**Phylogenetic analysis of 16S rRNA and HSP generated with the neighbor-joining method**. The phylogeny presented is based on the alignment of 1341 bp of the 16S rRNA and 201 residues of the HSP sequences. Theses consensus trees were rooted on the sequences of *Rhodococcus equi*, *Nocardia asteroides*, *Mycobacterium abscessus *and *Dietzia maris *(except for the HSP sequence not available for this species). Numbers next to each node indicate bootstrap values as a percentage of 1000 replicates. The bootstrap values below 50% are indicated by a slash (/). Each strain designation is followed by the corresponding Genbank accession number in parentheses. Asterisks indicate a Genbank accession numbers corresponding to a nucleotide sequence whose amino acid sequence was deduced.

A phylogenetic analysis was conducted on the nucleotide sequence of 16S rRNA gene, as well as on both the nucleotide and deduced amino acid sequences of the heat shock protein (HSP) from the isolate Bx-62986 and from other bacterial strains. Phylogenetic and molecular evolutionary analyses were conducted using the MEGA (Molecular Evolutionary Genetics Analysis) software version 4 [13]. Nucleotide sequences of the 16S rRNA gene as well as nucleotide and amino acid sequences of the HSP were downloaded from GenBank databases (except for the Bx-62986 isolate) and aligned using the multiple alignment options in CLUSTAL followed by minor manual adjustments. Phylogenetic trees were generated by the neighbor-joining method. Branching significance was estimated using bootstrap confidence levels by randomly resampling the data 1000 times with the referred evolutionary distance model. Both trees were similar. With regard to HSP sequences, higher bootstrap values were obtained with protein sequences than with nucleotide sequences (data not shown). Both trees were highly similar and the analysis placed most sequences into species-specific clusters (Figure 1). As expected, the isolate Bx-62986 was closely related to *T. tyrosinosolvens*. Globally, the high bootstrap values obtained with the HSP protein sequences suggest that this protein could be useful for phylogenetic analyses.

## Conclusion

Human infections with *T. tyrosinosolvens *are very rare with only six cases reported in the literature. In all of the cases, this bacterium was isolated in immunocompromized patients with gastric cancer [14] or chronic pulmonary infections [6] and in patients who were carriers of cardiac implants or intravascular catheters [15-17].

*Tsukamurella *species are strictly aerobic Gram-positive rods which can be easily misidentified as *Corynebacterium *species, *Nocardia *species or *Mycobacteria *species. The difficulties encountered in the identification of these bacteria were previously reported by Almuzara *et al *[18] who noted that the identification by the API Coryne bacterium System is limited and by Stanley *et al *[19] who failed in identifying them with GenoType. The difficulty in identifying the organism by phenotypic methods suggests that *T. tyrosinosolvens *and related species may well be underdiagnosed as a cause of pulmonary infections. In our case, blood culture helped to determine the etiology of pulmonary disease.

Sequence-based identification is an alternative method to identify clinical isolates that are either slow growers or difficult to identify by biochemical profiling. The 16S rRNA gene is the most frequent target used in clinical laboratories for molecular identification, but in some cases this tool can only help to identify the genus and it is not discriminative enough for speciation within the genus. Under such circumstances, sequences of essential genes such as *hsp *have been shown to be useful for the identification.

This report finally highlights the difficulty in identifying the genus *Tsukamurella*. There is currently no commercially available test for Gram-positive rods for the identification of Tsukamurella spp. Genotypic methods are therefore the logical solution to identify certain bacterial isolates. Unfortunately, they have not yet become routine methods in the laboratory. Nevertheless, they may be useful for epidemiological characterization of unusual causal agents of bacteremia in immunocompromized patients.

## Consent

Written informed consent was obtained from the patient for publication of this case report.

## Competing interests

The authors declare that they have no competing interests.

## Authors' contributions

AM and SD carried out the experimental design of the study. AM performed genotypic identifications and phylogenetic analyses. SD, OP and JM carried out bacterial cultures and phenotypic identifications. AM, TDTN, CD and JM co-drafted the manuscript. All authors read and approved the final manuscript.
